# Genome-Wide Identification and Expression of Chitinase Class I Genes in Garlic (*Allium sativum* L.) Cultivars Resistant and Susceptible to *Fusarium proliferatum*

**DOI:** 10.3390/plants10040720

**Published:** 2021-04-07

**Authors:** Mikhail A. Filyushin, Olga K. Anisimova, Elena Z. Kochieva, Anna V. Shchennikova

**Affiliations:** Research Center of Biotechnology, Institute of Bioengineering, Russian Academy of Sciences, Leninsky Ave. 33, bld. 2, 119071 Moscow, Russia; lelikanis@yandex.ru (O.K.A.); ekochieva@yandex.ru (E.Z.K.); shchennikova@yandex.ru (A.V.S.)

**Keywords:** garlic *Allium sativum* L., GH19 family, class I chitinases, biotic stress, *Fusarium* spp., gene structure, gene expression

## Abstract

Vegetables of the *Allium* genus are prone to infection by *Fusarium* fungi. Chitinases of the GH19 family are pathogenesis-related proteins inhibiting fungal growth through the hydrolysis of cell wall chitin; however, the information on garlic (*Allium sativum* L.) chitinases is limited. In the present study, we identified seven class I chitinase genes, *AsCHI1–7*, in the *A. sativum* cv. Ershuizao genome, which may have a conserved function in the garlic defense against *Fusarium* attack. The *AsCHI1–7* promoters contained jasmonic acid-, salicylic acid-, gibberellins-, abscisic acid-, auxin-, ethylene-, and stress-responsive elements associated with defense against pathogens. The expression of *AsCHI2*, *AsCHI3*, and *AsCHI7* genes was constitutive in *Fusarium*-resistant and -susceptible garlic cultivars and was mostly induced at the early stage of *F. proliferatum* infection. In roots, *AsCHI2* and *AsCHI3* mRNA levels were increased in the susceptible and decreased in the resistant cultivar, whereas in cloves, *AsCHI7* and *AsCHI5* expression was decreased in the susceptible but increased in the resistant plants, suggesting that these genes are involved in the garlic response to *Fusarium proliferatum* attack. Our results provide insights into the role of chitinases in garlic and may be useful for breeding programs to increase the resistance of *Allium* crops to *Fusarium* infections.

## 1. Introduction

Plants belonging to the *Allium* genus are widely cultivated for their culinary and medicinal properties [1]. Throughout their life cycle, onions (including garlic), like all other agricultural plants, are infected with fungi, in particular *Fusarium* spp., which are the most viable and destructive soil-dwelling crop pathogens causing *Fusarium* basal rot (*F. oxysporum* f. sp. *cepae*) and bulb rot (*F. proliferatum*) [2,3,4,5].

*F. oxysporum* f. sp. cepae is responsible for 60% of the world’s garlic crop losses. This species produces chlamydospores that can survive in the soil for many years and cause decease symptoms at all pre- and post-harvest stages, infecting the roots and basal plates [5]. The mycotoxigenic species *F. proliferatum* is able to colonize the roots of garlic plants, remaining a latent infection during growth, and develop rot during storage, affecting almost 30% of bulbs and producing a wide range of toxins [4]. Observed symptoms include dry brown necrotic spots, and sometimes white mycelium and water-soaked signs at the clove surface [4].

*Fusarium* infections are suppressed with fungicides, the use of resistant cultivars, and the intercropping of *Allium* spp., which is considered an effective method of *Fusarium* biological control [6,7]. It has been shown that *Fusarium* growth can be suppressed by antifungal volatiles such as 2-methyl-2-pentenal and organosulfur compounds and non-volatiles such as spirostanol, furostanol, and steroidal saponins produced by *Allium* roots [8,9,10,11,12], as well as by *Flavobacterium* spp. accumulated in *Allium* rhizospheres [13]. The mechanism underlying the fungicidal effects of *Allium* plants was addressed by RNA-seq analysis, which revealed 45 miRNAs involved in positive (miR164a, miR168a, and miR393) and negative (miR394) regulation of resistance to *Fusarium*, and identified differentially expressed genes encoding polysaccharide-hydrolyzing enzymes such as chitinases and β-glucanases implicated in *Allium* immune defense [5,14,15].

As the fungal cell wall is rich in chitin, a long-chain polymer of N-acetyl-β-D-glucosamine (GlcNAc) [16], chitinases (E.C. 3.2.1.14) play an important role in plant–fungi interactions. These glycoside hydrolases (GHs) inhibit the growth and spread of pathogenic fungi through the digestion of β-1,4-glycosidic bonds in the cell wall chitin [17], which results in the release of chito-oligosaccharides recognized by plant receptors containing a Lys motif (CEBiP and CERK1) and the induction of plant immune response [18,19]. Therefore, plant chitinases are considered pathogenesis-related (PR) proteins and have been classified into PR-3, PR-4, PR-8, and PR-11 families [20], which in turn are grouped into GH18 (PR-3 and PR-8) and GH19 (PR-3, PR-4, and PR-11) families according to protein structure and catalytic mechanism (carbohydrate-active enzymes (CAZy) database, http://www.cazy.org, accessed on 20 January 2021) [21]. GH18 mainly comprises animal, fungal, bacterial, and viral chitinases, whereas most plant chitinases belong to the GH19 family [22]. The few GH18 plant enzymes are class III and V endochitinases involved in the resistance to biotic and abiotic stresses [23,24,25,26,27]; however, their antifungal activity is much lower than that of GH19 chitinases [17,23,28], which act directly on the fungal cell wall chitin [29,30,31] and whose antifungal activity depends on the protein charge [32,33].

Based on domain architecture and sequence identity, the GH19 family is divided into classes I, II, IV, VI, and VII [29]. Class I members have an N-terminal chitin-binding domain (CBD), a linker region enriched in Thr, Pro, and Gly residues, a catalytic domain GH19, and a C-terminal extension (CTE). GH19 chitinases of classes I–VI lack some of these regions, whereas class VI enzymes are similar in size and structure to class I proteins, but form a distinct clade because of low sequence similarity in the N-terminal region [29].

Studies on purified chitinases or chitinase-overexpressing transgenic plants have demonstrated the antifungal activity of these enzymes which either directly hydrolyze fungal chitin or activate systemic acquired resistance in the host [34]. Thus, in both resistant and susceptible *Eucalyptus grandis* plants vacuolar targeted class I chitinase (GH19; Eucgr.I01495), as well as clusters of putative chitinase genes, particularly on chromosomes 3 and 8, are strongly upregulated in response to the fungal pathogen *Chrysoporthe austroafricana* [35], whereas extracts of transgenic *Nicotiana tabacum* overexpressing *Drosera rotundifolia* chitinase class I suppress the growth of another fungal pathogen, *Trichoderma viride* [36]. These studies point on the role of class I chitinases in antifungal plant immunity; however, the information on chitinases from *Allium* spp. is very limited. In onion (*A. cepa* L.), eight class I and one class VII chitinases have been characterized in silico and four of the class I enzymes are predicted to have a role in plant defense [37], whereas in garlic (*A. sativum* L.), only two chitinases are annotated in the NCBI GenBank (mRNA fragments M94105.1 and M94106.1; https://www.ncbi.nlm.nih.gov/ accessed on 6 April 2021).

Complete sequencing of the garlic genome and transcriptome [38] makes it possible to explore the genetic and functional signatures of chitinases in this species. In the present study, we identified in silico seven putative class I chitinase-encoding genes *AsCHI1–7* in the genome of *A. sativum* cultivar (cv.) *Ershuizao* and cloned their homologues from garlic cultivars resistant and susceptible to *Fusarium* rot. We analyzed the structure of genes, including their promoter regions, and their phylogeny. In addition, the expression of *AsCHI1–7* mRNA was determined in various tissues of garlic under normal conditions and in response to *F. proliferatum* infection. Based on the data obtained, we suggest a role for chitinases in garlic protection against fungal pathogens. Our results provide useful insights into the function of *A. sativum* chitinases, which can be used in breeding programs to increase the resistance to *Fusarium* in cultivated *Allium* spp.

## 2. Results

### 2.1. In Silico Genome-Wide Identification of Class I Chitinase Genes in A. sativum cv. Ershuizao

**Figure 1:**
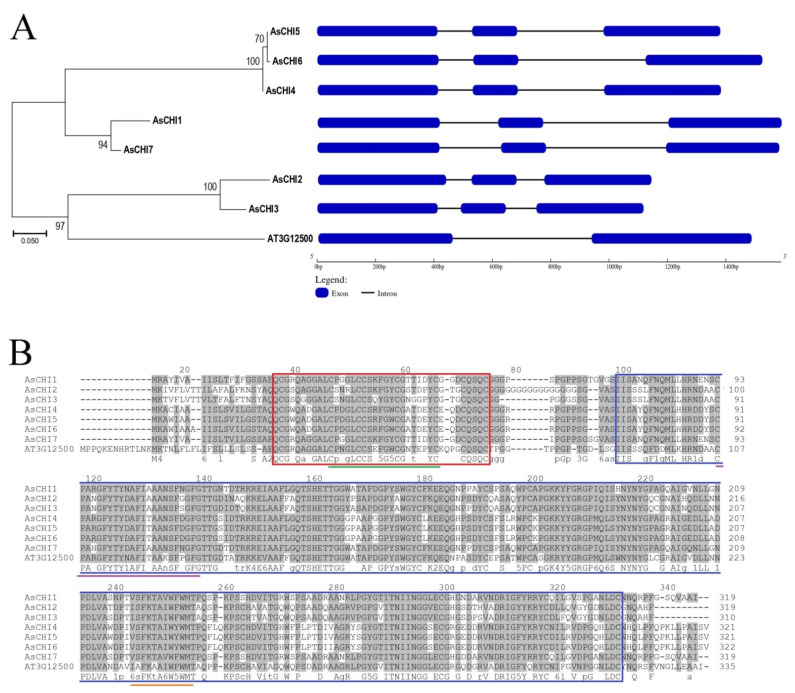
Structural and phylogenetic analysis of garlic chitinases. (**A**) Evolutionary relationship and exon–intron structures of class I chitinases from *Allium sativum* cv. Ershuizao (AsCHI1–7) and *Arabidopsis thaliana* (AT3G12500). The unrooted dendrogram is based on amino acid sequences. Analysis was performed using the maximum likelihood method. Percentages of replicate trees in which the associated sequences clustered together in the bootstrap test (1,000 replicates) are shown next to the branches. (**B**) Sequence alignment and functionally important sites of class I chitinases from *A. sativum* cv. Ershuizao and *A. thaliana*. The regions with 50–100% identity are shaded. The chitin-binding domain (CBD1) and glycoside hydrolase domain (GH19) are indicated by red and blue frames, respectively. CBD1 signature PS00026 (Cx(4,5)-CSx(2)-GxCGx(4)-[FYW]-C) is underlined in green, chitinase 19_1 signature PS00773 (Cx(4,5)-FY-[ST]-x(3)-[FY]-[LIVMF]-xAx(3)-[YF]-x(2)-F-[GSA])—in violet, and chitinase 19_2 signature PS00774 ([LIVM]-[GSA]-Fx-[STAG](2)-[LIVMFY]-W-[FY]-W-[LIVM])—in orange.

A total of seven complete sequences of class I chitinase genes were found in the genome and transcriptome of *A. sativum* cv. Ershuizao (PRJNA606385, PRJNA607255) and annotated as *AsCHI1–7* (*A. sativum* chitinases 1–7) (Table 1). The *AsCHI1* gene is located on chromosome 5 and the other six genes—on chromosome 6. The sizes of the identified genes range from 1118 to 1589 bp, and the coding sequences (CDSs) range from 933 bp in *AsCHI3* to 969 bp in *AsCHI6* and consist of three exons (Table 1, Figure 1A).

Phylogenetic analysis of the AsCHI protein sequences revealed three main clades: the first comprising closely related AsCHI4, AsCHI5, and AsCHI6 (99.1–99.4% identity), the second—AsCHI1 and AsCHI7 (95.3% identity), and the third—AsCHI2 and AsCHI3 (90.6% identity with 30 variable residues) as well as the only known class I chitinase from *Arabidopsis thaliana* (AT3G12500) (Figure 1A).

### 2.2. Structural and Phylogenetic Analyses of Class I Chitinases Predicted in Garlic

The sizes of the translated AsCHI homologues were 310 (AsCHI3), 319 (AsCHI1, AsCHI2, and AsCHI7), 321 (AsCHI4, and AsCHI5), and 322 (AsCHI6) amino acids (aa) (Table 1). The predicted physicochemical properties of AsCHI proteins are presented in Table 2. AsCHI aliphatic indexes showing the relative number of hydrophobic residues were from 52.73 to 63.42 and AsCHI hydrophobicity indexes (grand average hydropathy—GRAVY) calculated as the sum of hydrophobicity values of all residues divided by sequence length were negative, indicating the hydrophilic nature of the translated proteins.

Similar to other GH19 class I chitinases, putative AsCHI homologues contained a conserved CBD1 with the CHIT_BIND_I_1 signature (PS00026; C-x(4,5)-C-C-S-x(2)-G-x-C-G-x(4)-[FYW]-C) and the GH19 catalytic domain (PF00182) with two active site signatures: CHITINASE_19_1 (PS00773; C-x(4,5)-F-Y-[ST]-x(3)-[FY]-[LIVMF]-x-A-x(3)-[YF]-x(2)-F-[GSA]) and CHITINASE_19_2 (PS00774; [LIVM]-[GSA]-F-x-[STAG](2)-[LIVMFY]-W-[FY]-W-[LIVM]) (Figure 1B, Table 2). In addition, the GH19 domain contained a highly conserved motif [FHY]-G-R-G-[AP]-x-Q-[IL]-[ST]-[FHYW]-[HN]-[FY]-N-Y specific for GH19 chitinases [39] and N-terminal motifs MLXXR, XFYTYX, AFXXAA, FXXTGX, and TSH characteristic for class I chitinases [40].

The size of CBD1 (38 aa) was the same in all AsCHI proteins and those of the GH19 domains were 232 aa (AsCHI1, AsCHI2, AsCHI3, and AsCHI7) and 233 aa (AsCHI4, AsCHI5, and AsCHI6). N-terminal signal peptides (19–21 aa) with the predicted cleavage site SxA-(Q/E)Q were detected in all AsCHI proteins (Table 2).

**Figure 2:**
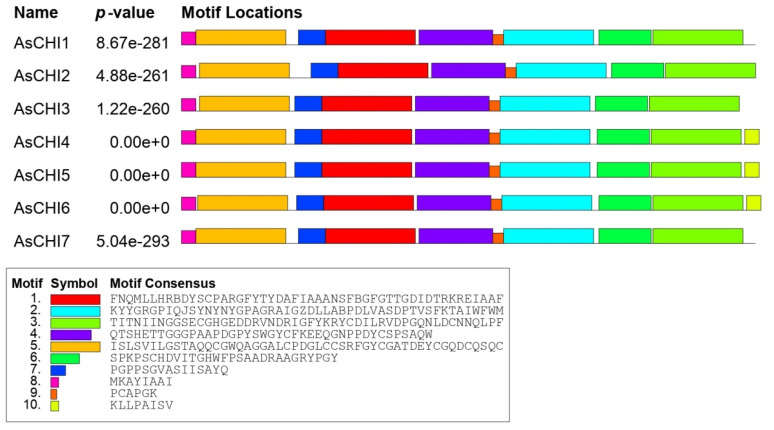
Distribution of conserved motifs in AsCHI proteins of *A. sativum* cv. *Ershuizao*. Analysis was performed using Multiple Em for Motif Elicitation (MEME) 5.3.0. The length of each box corresponds to that of the motif.

Multiple Em for Motif Elicitation (MEME)-based analysis revealed 10 conserved motifs in putative AsCHI enzymes, including motifs 5 (CBD1) and 8 (signal peptide). Motif 10 was characteristic for the C-terminus of clade 1 proteins AsCHI4, AsCHI5, и AsCHI6 (Figure 1A and Figure 2).

The total ratio of basic (Arg, His, and Lys) to acidic (Asp and Glu) residues in putative AsCHI proteins was 27/17 (AsCHI1), 24/18 (AsCHI2), 19/19 (AsCHI3), 34/30 (AsCHI4, 34/31 (AsCHI5), 34/31 (AsCHI6), and 26/23 (AsCHI7). Considering the pI value determined for each studied chitinase (Table 2), it can be proposed that AsCHI1 and AsCHI2are basic enzymes, while the rest of the proteins are acidic rather than basic.

Functional annotation in gene ontology (GO) terms predicted that the AsCHI proteins had chitinase activity and chitin-binding ability, and were involved in the catabolism of chitin, cell wall macromolecules, polysaccharides and carbohydrates, and in plant response to fungal infection (Table 2).

In the NCBI database of non-redundant protein sequences, AsCHI1 had the highest degree of identity with chitinases of *Cocos nucifera* (EHA8588849.1; 76%) and *Chimonanthus praecox* (ACN55075.1; 75%), AsCHI2–with chitinases of *Ananas comosus* (CAD1821239.1, OAY84822.1; 78%) and *C. nucifera* (EHA8588849.1; 77%), AsCHI3—with chitinases of *Elaeis guineensis* (XP_010941404.1; 77%) and *C. nucifera* (EHA8588849.1; 77%), AsCHI4-6—with chitinases of *E. guineensis* (XP_010941401.1; 68%), *Phoenix dactylifera* (XP_026656006.2; 66%), and *Acacia mangium* (BAO45893.1; 66%), and AsCHI7—with chitinases of *C. nucifera* (EHA8588849.1; 78%), *E. guineensis* (XP_010941404.1; 76%), and *Cinnamomum micranthum* (RWR90612.1; 76%).

### 2.3. In Silico Analysis of the Expression of A. sativum Class I Chitinases

Next, we analyzed the expression of class I chitinase genes in various tissues (roots, bulbs, pseudostem, leaves, buds, flowers, and sprouts) of *A. sativum* cv. Ershuizao based on transcriptomics data (PRJNA607255 and GSE145455 [38]). The analysis of gene expression in bulbs was more detailed than for other tissues, and included eight developmental stages (from 192 to 227 days old), since this underground part of the plant is most susceptible to *Fusarium* infection with visible symptoms. A less detailed analysis of other tissue types, in addition to the above, is due to the presence of transcriptome data for these tissues only for one stage [38].

**Figure 3:**
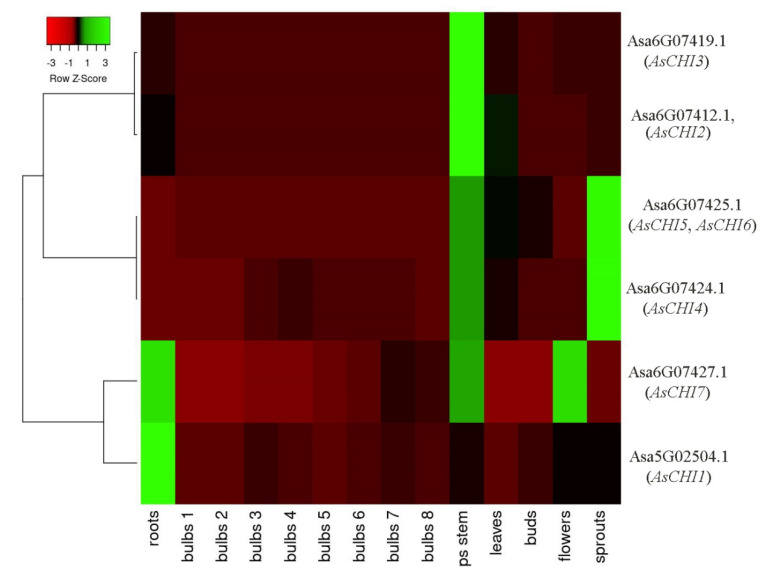
Heatmap of *AsCHI1–7* mRNA expression in *Allium sativum* cv. *Ershuizao* tissues. Gene transcription was analyzed in roots, bulbs (1, 2, 3, 4, 5, 6, and 7 correspond to 192-, 197-, 202-, 207-, 212-, 217-, 222-, and 227-day-old bulbs), pseudo (ps) stems, leaves, buds, flowers, and sprouts. Colors red to green indicate a gene expression gradient from low to high.

Figure 3 shows that *AsCHI2*/*AsCHI3* and *AsCHI4*–*6* had a similar expression pattern within each clade, whereas those of *AsCHI1* and *AsCHI7* were rather different. *AsCHI4*–*6* showed maximum expression in the pseudostem and shoots, and *AsCHI2*/*AsCHI3*—in the pseudostem. The *AsCHI1* gene was strongly expressed in the roots and *AsCHI7*—in the roots, pseudostem, and flowers.

During bulb development, the expression of *AsCHI2*, *AsCHI3*, *AsCHI5*, and *AsCHI6* was quite weak, whereas that of *AsCHI4* showed a bell shape, increasing up to stage 4 and then decreasing. The expression of *AsCHI7*, which was almost absent at stages 1 and 2, gradually increased, reaching a maximum at stage 7, whereas that of *AsCHI1* peaked at stage 3 (Figure 3). It should be noted that all genes, except *AsCHI1*, had a high mRNA expression level in the pseudostem, whereas only *AsCHI1* and *AsCHI7* were strongly expressed in the roots.

### 2.4. Promoter Analysis of Garlic Class I Chitinase Genes

The *AsCHI1–7* sequences upstream of the initiation codon (~1.0 kb), which predictably included the promoter and 5′UTR, were amplified and analyzed for hormone- and stress-responsive elements. The search for sites important for gene transcription showed that the *AsCHI1–7* regulatory regions contained 13 hormone-responsive elements (ABRE, ABRE3a, ABRE4, CARE, AUXRR-core, TGA-box, TGA-element, CGTCA-motif, TCA-element, TATC-box, P-box, GARE-motif, and ERE), and 12 stress-responsive elements (ARE, DRE1, DRE core, LTR, MBS, STRE, F-box, AT-rich sequence, TC-rich repeats, W-box, Wun-motif, and WRE3) (Table 3). Among them, four elements (methyl jasmonate (MeJA)-responsive CGTCA motif, ethylene (ET) responsive ERE, anaerobic induction essential ARE, and stress responsive STRE) were common for all analyzed genes which, however, differed in their numbers.

*AsCHI1* contained six abscisic acid (ABA)-responsive elements (ABRE), whereas the other genes contained one or two; ABRE3a and ABRE4 as well as dehydration-responsive DRE1 were found only in the *AsCHI1* gene (Table 3).

AT-rich sequence (maximal elicitor-mediated activation) was present only in *AsCHI2*, whereas ABA-responsive CARE and auxin-responsive TGA-box were found in *AsCHI2* and *AsCHI3*, and defense and stress responsive TC-rich repeats—in *AsCHI6* and *AsCHI7*. WRE3 involved in wound and pathogen responses was detected in *AsCHI2*, *AsCHI5*, and *AsCHI7*, and salicylic acid (SA)-responsive elements were found in *AsCHI2*, *AsCHI3*, and *AsCHI7* (Table 3).

There were no ABA- and auxin-responsive elements in *AsCHI6*, whereas gibberellin (GA)-responsive elements were absent in *AsCHI1* and *AsCHI7*, and dehydration-responsive elements—in *AsCHI3*, *AsCHI4*, and *AsCHI5*. Low temperature-responsive elements were absent in *AsCHI2*, *AsCHI5*, and *AsCHI7*, drought- and salt/heavy metals-responsive elements—in *AsCHI3-7*, and W-box (fungal elicitor and wound responsive)—in *AsCHI4* and *AsCHI7* (Table 3).

### 2.5. Class I Chitinase Gene Expression in cv. Sarmat and Strelets Infected with F. proliferatum

Data on chitinase genes obtained for cv. *Ershuizao* were used to elucidate the possible roles of the identified genes in two garlic cultivars known to be resistant and susceptible, respectively, to *Fusarium* infection.

**Figure 4:**
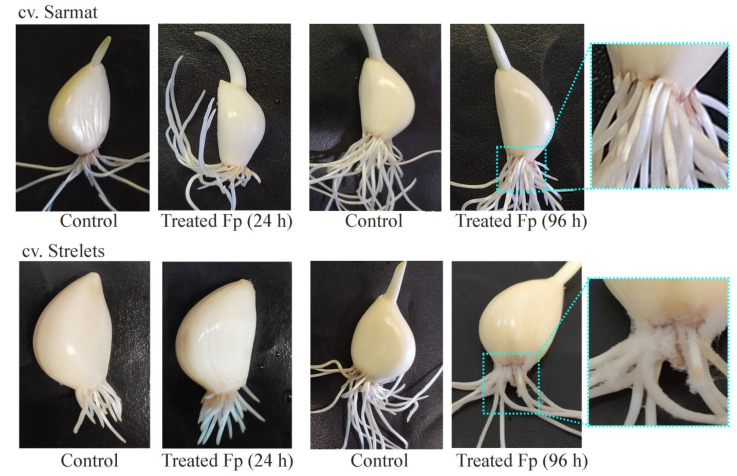
Symptoms of *Fusarium* rot disease in infection-resistant (Sarmat) and -susceptible (Strelets) garlic cultivars. Cloves were soaked in the suspension of *F. proliferatum* (Fp) conidia for 5 min and analyzed after 24 and 96 h; control cloves were treated with distilled water.

Garlic cv. Sarmat and Strelets resistant and susceptible to *Fusarium* rot, respectively, were infected with *F. proliferatum*, and analyzed for disease symptoms after 24 and 96 h. In cv. *Sarmat*, there were no symptoms of infection, whereas in cv. *Strelets*, the roots showed white fungal mycelium cover after 96 h (Figure 4). White mycelium is the reported symptom of *Fusarium* rot. Consequently, the external growth of the pathogen is observed only in the case of root colonization and indicates the sensitivity of the cultivar to this infection, while the tissues of the resistant cultivar are able to suppress the growth of fungi.

As sequences of some chitinase genes were very similar, common primer pairs were used to amplify *AsCHI4–6* and *AsCHI1*/*AsCHI7*. In these cases, the resulting products were sequenced to determine preferentially expressed genes.

**Figure 5:**
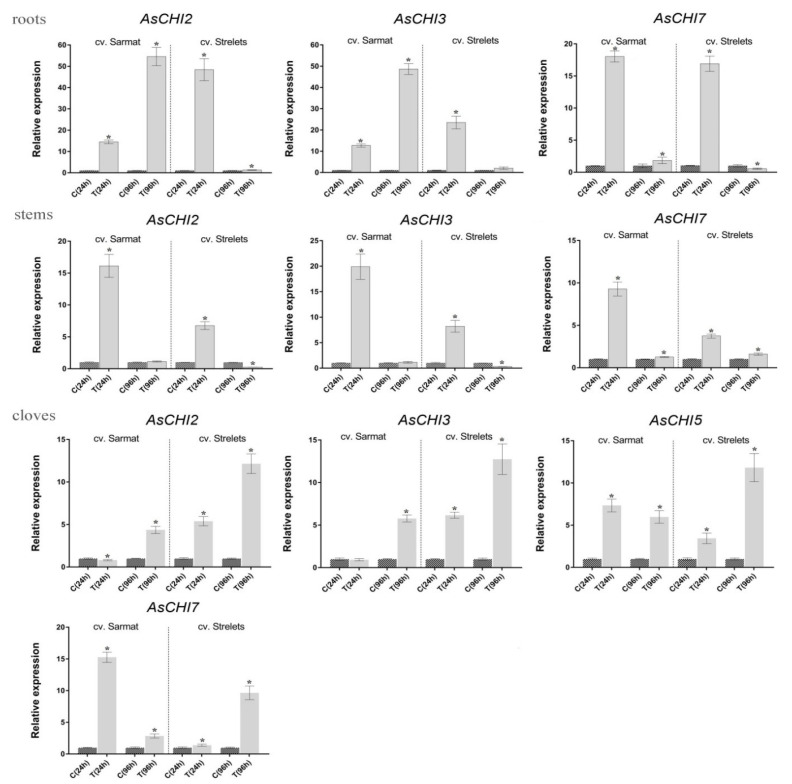
Expression of the *AsCHI* genes in roots, stems, and cloves of *Allium sativum* cv. *Sarmat* and *Strelets* after 24 and 96 h of *Fusarium proliferatum* infection. The data were normalized to *GAPDH* and *UBQ* mRNA levels and presented as the mean ± SE (*n* = 3) compared to control taken as 1. * *p* < 0.05 compared to uninfected control.

**Figure 6:**
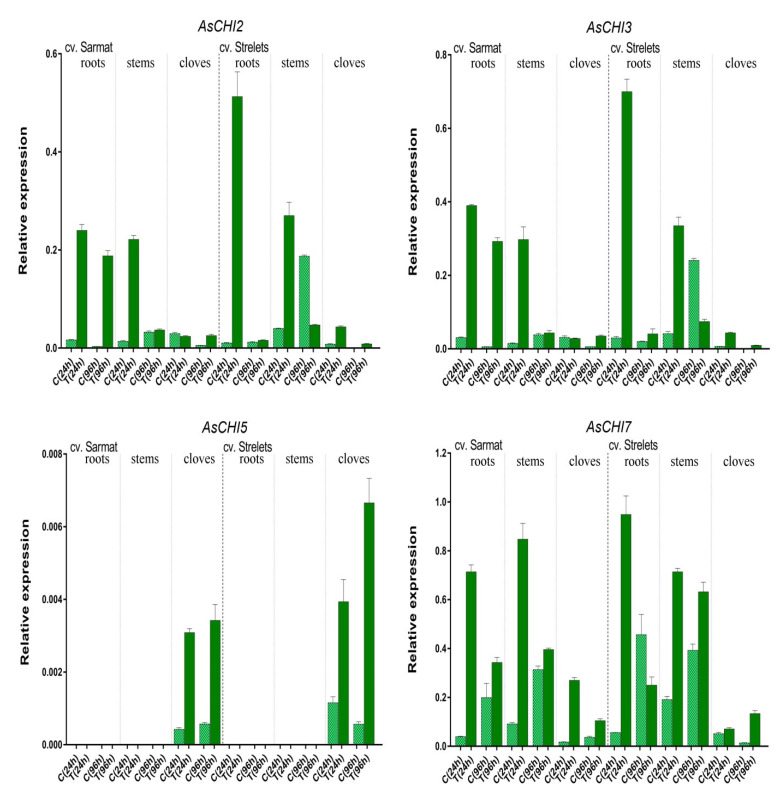
Expression of the *AsCHI* genes in roots, stems, and cloves of *Allium sativum* cv. Sarmat and Strelets: in control (C) and treated (T; after 24 and 96 h of *Fusarium proliferatum* infection) plants. The data were normalized to mRNA levels of *GAPDH* and *UBQ* genes and presented as the mean ± SE (*n* = 3).

Analysis of *AsCHI1-7* expression in roots, stems, and cloves of infected and control plants revealed that *AsCHI1*/*AsCHI7*, *AsCHI2*, and *AsCHI3* were transcribed in all analyzed organs, whereas *AsCHI4*–*6*—only in cloves (Figure 5 and Figure 6). In Sarmat roots, the chitinase genes were significantly upregulated at 24 and 96 h after infection and in Strelets roots, the upregulation was observed only at 24 h, whereas at 96 h the expression of some genes was decreased (*AsCHI1*/*AsCHI7*), slightly increased (*AsCHI2*), or was the same (*AsCHI3*) compared to the control (Figure 5). In the stems, the activation of class I chitinase genes was observed in both cultivars at 24 h, but in cv. *Sarmat* it almost doubled that in cv. *Strelets*; however, at 96 h the expression of all *AsCHI* genes was significantly downregulated. In the cloves, the transcription of the analyzed genes was overall higher in all infected samples compared to the control. The mRNA expression of *AsCHI2* and *AsCHI3* increased with time; in cv. *Strelets*, it markedly exceeded that in cv. *Sarmat* where the expression level at 24 h was almost the same as in the control. However, the transcription of *AsCHI1*/*AsCHI7* and *AsCHI4–6* had different dynamics in the two cultivars, increasing with time in cv. *Strelets* and decreasing in cv. *Sarmat* (Figure 5).

In control plants, the expression of the *AsCHI* genes was also time-dependent (Figure 6). In both analyzed cultivars, *AsCHI1*/*AsCHI7* transcription increased from 24 to 96 h, except for cv. *Strelets* cloves, where it decreased. Compared to 24 h, at 96 h *AsCHI2* and *AsCHI3* were upregulated in the stems but downregulated in the roots and cloves, whereas *AsCHI4*–*6* was slightly upregulated in the cloves of cv. *Sarmat* but significantly downregulated in those of cv. *Strelets*. In the stems of uninfected cv. *Strelets*, the levels of *AsCHI2*, *AsCHI3*, and *AsCHI1*/*AsCHI7* expression were higher than in those of cv. *Sarmat* (Figure 6).

### 2.6. Expression and Characterization of AsCHI CDSs in A. sativum cv. Sarmat and Strelets

To determine which genes of *AsCHI1*/*AsCHI7* and *AsCHI4–6* clades were expressed in the tissues of the two garlic cultivars, we performed sequencing of PCR-amplified products, which revealed that only *AsCHI7* and *AsCHI5* were transcribed in the roots, stems, and cloves of both cultivars.

CDSs of *AsCHI2*, *AsCHI3*, *AsCHI5*, and *AsCHI7* (Table 1) were amplified from the cDNA of garlic cv. Sarmat and Strelets using 5′- and 3’- UTR-specific primers and sequenced; the data have been deposited in the NCBI GenBank (Table 4). The sequences of *AsCHI* genes from the two cultivars were almost identical to those from cv. *Ershuizao* (Table 1), except for a few single nucleotide polymorphisms: 450A > T, 520T > C, 816C > T, and 940T > C (*AsCHI2*), 141T > C, 486T > A, 696G > A, 712A > G, 777C > T, 891C > A, and 915C > A (*AsCHI3*), 757G > A and 765C > G (*AsCHI5*, cv. Sarmat only), and 198A > G, 330A > T, 414C > T, 699A > G, 834T > C, and 901G > A (*AsCHI7*).

AsCHI2, AsCHI3, AsCHI5, and AsCHI7 proteins did not differ in size from cv. *Ershuizao chitinases* but contained the following residue substitutions: neutral S174P (AsCHI2), T238A (AsCHI3), and V253I (AsCHI5; cv. *Sarmat*), and radical G301S (AsCHI7).

### 2.7. Analysis of Promoters in Class I Chitinase Genes Differentially Expressed in cv. Sarmat and Strelets after F. proliferatum Infection

We next amplified and analyzed the promoters (~1.0 kb before the start codon) of the *AsCHI2, AsCHI3*, and *AsCHI7* genes, which were differently regulated in cv. *Sarmat* and *Strelets* in response to *F. proliferatum* infection. The *AsCHI2* promoter differed from the corresponding cv. *Ershuizao* sequence by four nucleotide substitutions, and between Sarmat and Strelets sequences by 1 bp indel at position −451 relative to cv. *Ershuizao* *AsCHI2* promoter.

The *AsCHI3* promoter differed from the corresponding cv. *Ershuizao* sequence by nine nucleotide substitutions and three indels (1, 3, and 20 bp). The 20 bp indel (TTGCTGACGTGAGAAAGGCA) was present at position −365 in cv. *Sarmat* and *Strelets* but at position −874 in cv. *Ershuizao*, which can be explained by inaccuracies in the assembly of the *A. sativum* genome. The *AsCHI7* promoter was identical in both cv. *Sarmat* and *Strelets*, and differed from the corresponding cv. *Ershuizao* sequence by 12 SNPs and two indels (2, and 3 bp).

The search for sites important for *AsCHI2, AsCHI3*, and *AsCHI7* transcription in cv. *Sarmat* and *Strelets* revealed the same cis-regulatory elements as in the *AsCHI2* promoter of the corresponding genes in cv. *Ershuizao* (Table 3). Compared to cv. *Ershuizao*, the *AsCHI3* promoter of cv. *Sarmat* and *Strelets* acquired two additional elements, ABRE and P-box, at position −365 in cv. *Ershuizao* *AsCHI3* promoter and lost the W-box element; the *AsCHI7* promoter of cv. *Sarmat* and *Strelets* acquired additional TC-rich repeat.

## 3. Discussion

The genus *Allium* (family Amaryllidaceae, order Asparagales) comprises 971 species, including economically important cash crops such as diploid garlic *A. sativum*, which has one of the largest genomes (16.24 Gb vs. 157 Mb of *A. thaliana* genome) among vegetables [38,41,42]. Significant pre- and post-harvest losses of garlic regularly occur worldwide because of diseases caused by soil fungi of the *Fusarium* genus [43]. The antifungal activity of class I chitinases indicate their role in the mechanism of garlic resistance to *Fusarium* rot, which can make these enzymes a promising target in crop breeding programs.

In the present study, we identified and characterized seven class I chitinase genes in the *A. sativum* cv. *Ershuizao* genome [38]); among them, four were amplified from garlic cv. *Sarmat* and *Strelets* resistant and susceptible to *Fusarium* rot, respectively. All the genes were orthologues of the only known *A. thaliana* class I chitinase gene (PR3; OAP02400.1), suggesting redundancy or, conversely, functional divergence among *AsCHI1–7*, such as involvement in differential responses to various stresses and pathogens. Analysis of chromosomal localization showed that the garlic genome contained two clusters of tandemly arranged chitinase genes, *AsCHI4–7* and *AsCHI*/*AsCHI2*, which may be a result of tandem duplication. All *AsCHI1–7* genes had only two introns (Figure 1), which is similar to the structure of *Brassica rapa* chitinase genes shown to be induced at the early stage of clubroot infection [44]. This finding confirms the hypothesis about the rapid regulation of stress-related genes during stress, which is considered to be partly due to a low number of introns [45,46].

All putative AsCHI1–7 proteins contained chitin-binding and catalytic domains, suggesting functional conservation of garlic class I enzymes in regard to chitin hydrolysis. The presence of N-terminal signal peptides indicates the secretion of mature proteins, which is supported by GO analysis predicting the involvement of AsCHI1–7 in the secretory pathway (Table 2) and is in agreement with the subcellular localization of *A. thaliana* chitinases [47]. The structure of putative AsCHI1–7 chitinases may also indicate participation in the response to fungal pathogens, as apoplastic chitinases were shown to be induced at the early stage of infection and block the intercellular hyphae growth, acting together with pathogen elicitors to initiate downstream defense pathways [48].

The expression of chitinase genes is triggered by phytohormones (e.g., MeJA and SA) and pathogen attack [49,50,51]. Thus, 20 hormone- and stress-related cis-regulatory elements were found in the promoters of the *Cucumis sativus* chitinase genes [52]. Cis-acting elements associated with response to SA, MeJA, auxin, ET, and GA as well as TC-rich repeats involved in defense and stress response were identified in the promoters of *B. rapa* class I chitinase genes [44]. Similar to *C. sativus*, but unlike *B. rapa*, the *AsCHI1–7* promoters contained elements associated with the response to ABA as well as those related to immune defense and response to elicitors and various stresses such as anaerobic conditions, dehydration, low temperature, salinization, heavy metals, and wounding (Table 3). Among the detected stress response elements, the fungal elicitor-responsive W-box was reported to be associated with the induction of chitinase gene expression by a transcription factor implicated in host antifungal defense [53]. It is assumed that other stress-responsive elements such as STRE and WUN-motif mediate pathogen- and/or elicitor-inducible transcription of chitinase genes [52,54].

In plants, pathogen attacks induce systemic or local acquired resistance associated with antagonistic SA and JA defense signaling, respectively [21,55]. The SA pathway leads to the accumulation of PR1, PR2, and PR5 proteins, whereas the JA pathway—to that of PR3, PR4 and PR12 proteins [21]. Thus, in garlic an attack by necrotrophic *Fusarium* fungi should trigger JA signaling, including the activation of class I chitinases belonging to PR3, PR4, and PR11 families, because the *AsCH1–7* promoters contain MeJA-responsive elements, indicating the participation of AsCHI1–7 enzymes in local acquired resistance. However, SA-responsive elements found in the promoters of *AsCHI2*, *AsCHI3*, and *AsCHI7* genes may also suggest their role in systemic acquired resistance.

ET is responsible for the defense against necrotrophic pathogens [55], and the presence of the ET-responsive element ERE found in all *AsCHI1–7* promoters (Table 3) suggests the possibility of their induction by ET after *Fusarium* attack.

Infection with pathogens can also modulate ABA homeostasis in host plants [44]. For example, infection with necrotrophic pathogen *Botrytis cinereal* promotes ABA biosynthesis in *Arabidopsis* [56], whereas increased ABA biosynthesis in tomatoes in response to bacterial attack inhibits pathogen penetration through stomatal closure [57]. Four *AsCHI* genes contain ABA-responsive promoter elements; among them, *AsCHI1* contains 14 (Table 3).

An important role in mediating biotic stress response is played by the crosstalk of JA with auxins and GAs, which promotes plant growth [58]. Among the *AsCHI* gene promoters, GA-responsive elements were absent only in *AsCHI1* and *AsCHI7*; however, the *AsCHI7* promoter had six auxin-responsive elements. Thus, *AsCHI1* and *AsCHI7* may not participate in GA-mediated defense, but may be involved in ABA- and auxin-mediated response, respectively, to *Fusarium*.

A possible role of the identified chitinases in the protection of garlic against *Fusarium* was revealed by examining the *AsCHI1–7* expression pattern in tissues of control and infected bulbs of garlic cultivars differing in the resistance to *Fusarium* basal rot. The results indicated that three genes, *AsCHI1*, *AsCHI4*, and *AsCHI6*, were not functional in cv. *Sarmat* and *Strelets*, since their mRNA could not be detected irrespectively of *F. proliferatum* infection; in contrast, *AsCHI4* and *AsCHI1* transcripts were observed in the pseudostem and roots, respectively, of cv. *Ershuizao* (Figure 3). It is possible that these genes are expressed in a cultivar-specific manner and involved in responses to pathogens other than *Fusarium* spp. *AsCHI2*, *AsCHI3*, and *AsCHI7* were transcribed constitutively, whereas *AsCHI5* expression was specific to cloves ((Figure 6).

All genes were differentially regulated by infection, which is consistent with the induction of chitinase expression in plants under biotic stresses [21]. In both resistant and susceptible cultivars, *AsCHI2*, *AsCHI3*, and *AsCHI7* genes showed early induction, except for *AsCHI2* and *AsCHI3* in cv. *Sarmat* cloves, where these genes were upregulated later (Figure 5). The most pronounced difference between the response of the resistant and susceptible cultivars to *Fusarium* was the time-dependent upregulation of *AsCHI2* and *AsCHI3* expression in cv. *Sarmat* roots and their downregulation in cv. *Strelets* roots. Furthermore, *AsCHI7* and *AsCHI5* mRNA levels decreased in the cloves of cv. *Sarmat* while increasing in those of cv. *Strelets* (Figure 5). These results suggest that the expression of *AsCHI2*, *AsCHI3*, *AsCHI5*, and *AsCHI7* may underlie the difference between resistant and susceptible cultivars in the response to *Fusarium* infection. The identical promoter sequences of these genes in resistant and susceptible garlic plants suggest that their expression after infection is controlled at the level of the activity of transcriptional regulators.

The results obtained can be useful for breeding programs with the aim of increasing the resistance of onion crops to *Fusarium* infections, since chitinases, which have antifungal activity and are differently expressed in cultivars resistant and susceptible to fungi, may be involved in plant defense from pathogens. The protective effect can be achieved by homologous and heterologous overexpression of chitinase genes, which has already been tried by many researchers. For example, overexpression of the *Momordica charantia* chitinase gene *McCHIT1* in rice (*Oryza sativa* subsp. *indica*) significantly increased the resistance of transgenic lines to Sheath blight compared to wild-type plants [59]. Heterologous overexpression of the *Coniothyrium minitans* chitinase gene *CmCH1* led to the upregulation of defense-related genes and enzymes in soybean plants, and thus enhanced their resistance to *Sclerotinia sclerotiorum* infection [60].

## 4. Materials and Methods

### 4.1. In Silico Identification and Structural Characterization of A. sativum Class I Chitinase Genes

The search for class I chitinase genes was performed in the *A. sativum* cv. Ershuizao transcriptome sequence (NCBI accession number: PRJNA607255) and whole-genome shotgun contigs (NCBI accession number: PRJNA606385) [38]; partial sequences of *A. sativum* class I chitinases (M94105.1 and M94106.1) were used as references. We selected sequences that contained start and stop codons and full-length CBD1 and GH19 domains characteristic of class 1 chitinases but did not have premature stop codons in CDSs.

Multiple sequence alignment and structural and phylogenetic analyses of chitinase genes and encoded proteins were conducted with MEGA 7.0.26 [61]. To predict exon–intron structures, chitinase genes and CDSs were analyzed with GSDS v2.0 [62]. Predicted proteins were characterized by molecular weight, isoelectric point (pI), aliphatic index and grand average hydropathy (GRAVY) (ExPASy ProtParam; https://web.expasy.org/protparam/; accessed on 10 November 2020), conserved domains, sites, and motifs (NCBI-CDD, https://www.ncbi.nlm.nih.gov/cdd, accessed on 10 November 2020; UniProt, https://www.uniprot.org/, accessed on 10 November 2020; and Multiple Em for Motif Elicitation (MEME 5.1.1) [63], http://meme-suite.org/tools/meme, accessed on 1 December 2020), biological processes and molecular functions (PANNZER2; http://ekhidna2.biocenter.helsinki.fi/sanspanz/, accessed on 1 December 2020), subcellular localization (BaCello; http://gpcr2.biocomp.unibo.it/, accessed on 1 December 2020), the functional importance of residue substitutions (PROVEAN; [64]), and signal peptide cleavage sites (SignalP 5.0; http://www.cbs.dtu.dk/services/SignalP/, accessed on 1 December 2020). The phylogenetic dendrogram was constructed based on protein sequences using the MEGA 7.0.26 (maximum likelihood (ML) method); confidence for tree topologies was estimated by bootstrap values of 1000 replicates.

### 4.2. In Silico mRNA Expression Analysis

The expression of class I chitinase genes in garlic tissues was determined using *A. sativum* cv. Ershuizao RNA-Seq data (FPKM; ID: PRJNA607255) [38] and visualized using Heatmapper [65].

### 4.3. Gene Identification

To amplify full-length chitinase genes from garlic cultivars, gene-specific primers were designed based on *A. sativum* cv. Ershuizao transcriptomic data (NCBI accession number: PRJNA607255) (Table 5); manual revision of sequence polymorphisms and additional evaluation were performed using Primer3 (http://frodo.wi.mit.edu/primer3/, accessed on 15 October 2020). Genomic DNA was isolated from young leaves of a single plant of each cultivar accession as previously described [66] and used as a template (100 ng) for PCR amplification at the following conditions: initial denaturation at 94 °C for 5 min, 35 cycles of denaturation at 94 °C for 30 s, primer annealing at 55 °C for 30 s, and extension at 65 °C for 2 min, and final extension at 65 °C for 5 min. The amplified PCR products of the expected size were purified by using the QIAEX^®^ II Gel Extraction kit (QIAGEN, Hilden, Germany), cloned in the pGEM^®^-T Easy (Promega, Madison, WI, USA), and sequenced (3–5 clones for each accession) on ABI Prism 3730 DNA Sequencer (Applied Biosystems, Waltham, MA, USA) using the designed primers.

### 4.4. Plants, Fungi, and Fusarium Infection Assay

Accessions of garlic cv. Sarmat and Strelets resistant and susceptible to *Fusarium* rot, respectively, were kindly provided by the Federal Scientific Vegetable Center (Moscow region, Russia) and *F. proliferatum* was kindly provided by the Group of Experimental Mycology, Winogradsky Institute of Microbiology (Research Center of Biotechnology of the RAS, Moscow, Russia).

The choice of cultivars for comparative analysis was based not only on the difference in resistance to fungal diseases, but also on the similarity of other varietal morphological characteristics for the possible elimination of their influence on the experimental results. Both cultivars are winter garlic of Russian breeding. These are mid-season, shooting varieties: the leaf is green with a waxy bloom of medium intensity, leaf length/width is 50–51/2.0–2.3 cm; the bulb is round–flat, 65 g; the number of cloves is 5–7 (Strelets) or 7–11 (Sarmat); the structure of the cloves is simple; the color of dry scales is lilac–purple (Strelets) or lilac–white with anthocyanin streaks (Sarmat), leathery scales are brown (Strelets) or light pink (Sarmat), the flesh is white; productivity is 2.0 (Strelets) and 1.9 (Sarmat) kg m^−2^. Data on the resistance of cultivars to *F. oxysporum* infection were obtained from their originators (the Federal Scientific Vegetable Center, Moscow region, Russia).

The *F. proliferatum* strain was previously isolated from garlic bulbs (cv. Strelets); the pathogenicity test showed that the first signs of the decease appear on the surface of the treated cloves after five days of infection (Filyushin et al., Plant Desease, accepted 3 April 2021). Since this strain was identified in cv. Strelets, and, in addition, it was identified for the first time in Russia, it was decided to use this particular strain in the present study.

The number of cloves in a bulb (5–7 for cv. Strelets and 7–11 for cv. Sarmat) determined the number of cloves used per biological replicate in the *Fusarium* infection experiment. In total, six bulbs of each cultivar were used; six cloves from each bulb were processed and halved for control and experiment.

Cloves of each cultivar were sterilized in 70% ethanol for 3 min, rinsed with sterile water, placed in Petri dishes with wet filter paper, and incubated at +25 °C in the dark. After 36 h, active root growth was observed and half of the cloves of each cultivar were infected by soaking in *F. proliferatum* conidial suspension (~10^6^ conidia mL^−1^) for 5 min (the inoculation procedure was carried out according to [67]). Then, the infected cloves were transferred to fresh Petri dishes and incubated at +25 °C in the dark; uninfected cloves were used as the control. The experiment was performed in three biological replicates. Various tissues (roots, stems (basal plates), and cloves) were collected at 24 (tissues of three out of six treated and control cloves from each replicate) and 96 h (tissues of the remaining three treated and control cloves from each replicate) after inoculation, immediately frozen in liquid nitrogen, and stored at −80 °C. Expression was checked at 24 h and 96 h after inoculation, as it has been reported that the genes of certain pathogenesis-related proteins showed peak expression 1–3 days after inoculation with hemibiotrophic pathogens [52,68].

### 4.5. RNA Extraction and Quantitative Real-Time Reverse Transcription PCR (qRT-PCR)

Total RNA was extracted from individual roots, stems, and cloves (0.5 g of each tissue, pre-ground to powder in liquid nitrogen) using the RNeasy Plant Mini Kit (QIAGEN, Hilden, Germany), purified from genomic DNA (RNase free DNasy set; QIAGEN, Hilden, Germany), qualified by gel electrophoresis, and used for first-strand cDNA synthesis (GoScript Reverse Transcription System; Promega, Madison, WI, USA) with an oligo-dT primer. RNA and cDNA concentrations were quantified by fluorimetry (Qubit^®^ Fluorometer, Thermo Fisher Scientific, Waltham, MA, USA) and qRT-PCR was performed in a CFX96 Real-Time PCR Detection System (Bio-Rad Laboratories, Hercules, CA, USA) with 3.0 ng of cDNA, SYBR Green RT-PCR mixture (Syntol, Moscow, Russia), and specific primers (Table 5). The following cycling conditions were used: initial denaturation at 95 °C for 5 min and 40 cycles of denaturation at 95 °C for 15 s and annealing/extension at 60 °C for 40 s.

*AsCHI* gene expression was normalized using two reference garlic genes, *GAPDH* [69] and *UBQ* [70], and qRT-PCR results were statistically analyzed with Graph Pad Prism version 8 (GraphPad Software Inc., San Diego, CA, USA; https://www.graphpad.com/scientific-software/prism/, accessed on 12 December 2020). The data were expressed as the mean ± standard deviation (SE) based on three technical replicates of three biological replicates for each combination of cDNA and primer pairs. The unequal variance (Welch’s) *t*-test was applied to assess differences in gene expression; *p* < 0.05 was considered to indicate statistical significance.

### 4.6. Promoter and 5′-UTR Identification and Analysis

The regulatory regions of chitinase genes were amplified by PCR using specific primers designed based on *A. sativum* cv. Ershuizao transcriptome sequence (Table 5), purified, cloned, and sequenced. The search of specific cis-elements in promoters and 5′-UTRs (1.0 kb regions upstream of the initiation codon) was performed using the PlantCARE database, which provides an evaluation of cis-regulatory elements, enhancers, and repressors [71]; (http://bioinformatics.psb.ugent.be/webtools/plantcare/html/; accessed on 25 January 2021).

## 5. Conclusions

We identified and characterized seven genes encoding class I chitinases in *A. sativum* cv. Ershuizao and cloned *AsCHI2*, *AsCHI3*, *AsCHI5*, and *AsCHI7* homologues from two garlic cultivars resistant and susceptible to *Fusarium* rot. The chitinase gene promoters contained hormone- and stress-responsive elements, including those associated with responses to fungal pathogens and their elicitors, suggesting that the putative garlic chitinases participate in local acquired resistance, whereas AsCHI2, AsCHI3, and AsCHI7 may be involved in systemic acquired resistance. Upon *Fusarium* attack, all *AsCHI* genes can be induced by ET, whereas *AsCHI1* and *AsCHI7* may be activated by ABA and auxin, respectively. *AsCHI1–7* transcriptional profiling in resistant and susceptible garlic cultivars infected with *F. proliferatum* suggests that the expression of *AsCHI2*, *AsCHI3*, *AsCHI5*, and *AsCHI7* genes could define the difference in chitinase-mediated response to *Fusarium* infection between resistant and susceptible plants. Our results provide useful insights into the functions of class I chitinases in *A. sativum* and *Allium* plants in general, and may be used in breeding programs to increase the resistance of *Allium* crops to *Fusarium* infections.

## Figures and Tables

**Table 1:** Characteristics of predicted class I chitinase genes in the *Allium sativum* cv. *Ershuizao* genome (PRJNA606385).

Gene	Genomic Location (Strand)	Transcript ID in RNA-Seq Data [37]	Size (bp)	Number of Exons	CDS (bp)
*AsCHI1*	ch05: 744404486–744406074 (−)	Asa5G02504.1	1589	3	960
*AsCHI2*	ch06: 936506601–963507740 (+)	Asa6G07412.1	1140	3	960
*AsCHI3*	ch06: 937609134–937610251 (+)	Asa6G07419.1	1118	3	933
*AsCHI4*	ch06: 938113646–938115026 (−)	Asa6G07424.1	1381	3	966
*AsCHI5*	ch06: 938164705–938166085 (−)	Asa6G07425.1 *	1381	3	966
*AsCHI6*	ch06: 938218393–938219916 (−)		1524	3	969
*AsCHI7*	ch06: 938640561–938642142 (−)	Asa6G07427.1	1582	3	960

* *AsCHI5* and *AsCHI6* sequences were detected in a single 1797 bp transcript Asa6G07425.1 (1–963 bp—*AsCHI6*; 7–412 and 567–966 bp—*AsCHI5*).

**Table 2:** Characteristics of the predicted class I chitinases in *A. sativum* cv. *Ershuizao*. GRAVY: grand average hydropathy.

Protein Symbol	Size (aa)	MW (kDa)	pI	Location of Specific Regions (aa)			Aliphatic Index	GRAVY	Functional Annotation in Gene Ontology Categories		
				Signal Peptide	CBD1	GH19 Domain			Cellular Component	Biological Process	Molecular Function
AsCHI1	319	33.72	8.47	1–19	21–58	75–306	57.62	−0.304	Secretory pathway	chitin catabolic process (GO:0006032) cell wall macromolecule catabolic process (GO:0016998) defense response to fungus (GO:0050832) carbohydrate metabolic process (GO:0005975)	chitinase activity (GO:0004568) chitin binding (GO:0008061)
AsCHI2	319	33.43	7.39	1–21	23–60	82–313	52.73	−0.275	Secretory pathway	chitin catabolic process (GO:0006032) cell wall macromolecule catabolic process (GO:0016998) polysaccharide catabolic process (GO:0000272) defense response to fungus (GO:0050832)	chitinase activity (GO:0004568) chitin binding (GO:0008061)
AsCHI3	310	32.74	5.40	1–21	23–60	73–304	54.26	−0.246	Secretory pathway	chitin catabolic process (GO:0006032) cell wall macromolecule catabolic process (GO:0016998) polysaccharide catabolic process (GO:0000272) defense response to fungus (GO:0050832)	chitinase activity (GO:0004568) chitin binding (GO:0008061)
AsCHI4	321	35.17	6.05	1–19	21–58	73–305	62.99	−0.346	Secretory pathway	chitin catabolic process (GO:0006032) cell wall macromolecule catabolic process (GO:0016998) polysaccharide catabolic process (GO:0000272) defense response (GO:0006952) response to fungus (GO:0009620)	chitinase activity (GO:0004568) chitin binding (GO:0008061)
AsCHI5	321	35.26	5.88	1–19	21–58	73–305	62.99	−0.357	Secretory pathway	chitin catabolic process (GO:0006032) cell wall macromolecule catabolic process (GO:0016998) defense response (GO:0006952) response to fungus (GO:0009620) carbohydrate metabolic process (GO:0005975)	chitinase activity (GO:0004568) chitin binding (GO:0008061)
AsCHI6	322	35.34	5.88	1–20	22–59	74–306	63.42	−0.349	Secretory pathway	chitin catabolic process (GO:0006032) cell wall macromolecule catabolic process (GO:0016998) defense response (GO:0006952) response to fungus (GO:0009620) carbohydrate metabolic process (GO:0005975)	chitinase activity (GO:0004568) chitin binding (GO:0008061)
AsCHI7	319	33.79	6.19	1–19	21–58	75–306	60.06	−0.325	Secretory pathway	chitin catabolic process (GO:0006032) cell wall macromolecule catabolic process (GO:0016998) defense response to fungus (GO:0050832) carbohydrate metabolic process (GO:0005975)	chitinase activity (GO:0004568) chitin binding (GO:0008061)

**Table 3:** Regulatory elements found in silico in the promoter sequences of *Allium sativum* cv. *Ershuizao* class I chitinase genes. MeJA: methyl jasmonate.

Functional Description	Annotation	Motif	Number of Elements Found in Gene Promoters						
			*AsCHI1*	*AsCHI2*	*AsCHI3*	*AsCHI4*	*AsCHI5*	*AsCHI6*	*AsCHI7*
Hormone responsive	Cis-acting elements, involved in abscisic acid response	ABRE	6	1	2		1		
		ABRE3a	4						
		ABRE4	4						
		CARE		1	1				
	Cis-acting elements involved in auxin response	AUXRR-core	1				1		1
		TGA-box		1	1				
		TGA-element				1	1		5
	Cis-acting element involved in MeJA response	CGTCA-motif	1	1	5	2	7	2	1
	Cis-acting element involved in salicylic acid response	TCA-element		2	1				1
	Cis-acting element involved in gibberellin response	TATC-box		1					
		P-box			1	1			
		GARE-motif			1	2	1	1	
	Ethylene-responsive element	ERE	1	2	9	1	2	2	2
Stress responsive	Cis-acting element essential for anaerobic induction	ARE	4	3	1	8	2	3	2
	Dehydration-responsive element	DRE1	2						
		DRE core		1			1		1
	Cis-acting element involved in low temperature response	LTR	1		1	3		1	
	MYB-binding site involved in drought response	MBS	1	1					
	Stress-responsive element	STRE	4	1	2	4	5	1	1
	Salt and heavy metal stress response element	F-box	1	1					
	Maximal elicitor-mediated activation	AT-rich sequence		2					
	Cis-acting element involved in defense and stress responses	TC-rich repeats						1	1
	Fungal elicitor and wound responses	W-box	1	2	1		2	1	
	Wound-responsive element	Wun-motif		2	1		2	2	
	Wound and pathogen response	WRE3			1	1		1	

**Table 4:** Characteristics of *AsCHI* homologous genes identified in *Allium sativum* cv. *Sarmat* and *Strelets*. CDS: coding sequence.

Gene	cv. Sarmat					cv. Strelets				
	NCBI ID	Number of SNPs	Gene (bp)	CDS (bp)	Protein (aa)	NCBI ID	Number of SNPs	Gene (bp)	CDS (bp)	Protein (aa)
*AsCHI2*	MW770892	4	1141	960	319	MW770893	4	1141	960	319
*AsCHI3*	MW770894	7	1118	933	310	MW770895	7	1118	933	310
*AsCHI5*	MW770896	2	1397	966	321	MW770897	0	1397	966	321
*AsCHI7*	MW770898	6	1579	960	319	MW770899	6	1579	960	319

**Table 5:** List of primers for *AsCHI1-7* gene amplification, sequencing, and expression analysis.

Genes/Gene Groups	Primer Sequences (5′→3′)	Amplicon Size (bp)	Application
*AsCHI1* *AsCHI7*	ATAAAAGYGGTGGTACATTGC GTACATAAAACTCATRTGCGWA	~1100	Gene amplification and sequencing
*AsCHI2*	GTAGATRCAGTCCTRCTGCT ATATCATATGACGACTTCGC	~1100	
*AsCHI3*	GTAGATRCAGTCCTRCTGCT ATTGCACATGTATCATATGAGG	~1100	
*AsCHI4* *AsCHI5* *AsCHI6*	TAAAAGGAGAGGTACGCAC GTAATTATTGCAAGCATCGTAA	~1100	
*AsCHI2*	CTTTCCAGAAACCTGTGACT TGCAGCTGCTATGAAGGCA	~1000	Regulatory region amplification and sequencing
*AsCHI3*	GTAAATGAGCATGGGTAAGTTG GTATTGGCTGCAGCATAGC	~1000	
*AsCHI7*	AGCACCACCAGCTTGTCTA ATGAGAACCGCGTTGATCGT	~1000	
*AsCHI1* *AsCHI7*	GTACCACTGGGGATACCGAT CCCCATGAATATGGTCCATCG	114	qRT-PCR
*AsCHI2*	GGAACCACTGGAGACATCAATG GCCTTGTTCTTGCTTGAAGCAG	140	
*AsCHI3*	GGAACCACTGGAGACATCGATA GCCTTGTTCTTGCTTGAAGCAG	140	
*AsCHI4* *AsCHI5* *AsCHI6*	GGTACCACCGGGAGTATTGAC ACCCCATGAATATGGTCCACCT	116	

## Data Availability

Not applicable.

## References

[1] Mnayer D., Fabiano-Tixier A.S., Petitcolas E., Hamieh T., Nehme N., Ferrant C., Fernandez X., Chemat F. (2014). Chemical composition, antibacterial and antioxidant activities of six essentials oils from the Alliaceae Family. Molecules.

[2] Snowdon A. (1990). A Color Atlas of Post-Harvest Diseases of Fruits and Vegetables.

[3] Akhter A., Hage-Ahmed K., Soja G., Steinkellner S. (2016). Potential of Fusarium wilt-inducing chlamydospores, in vitro behaviour in root exudates and physiology of tomato in biochar and compost amended soil. Plant Soil.

[4] Gálvez L., Urbaniak M., Waśkiewicz A., Stępień Ł., Palmero D. (2017). Fusarium proliferatum—Causal agent of garlic bulb rot in Spain: Genetic variability and mycotoxin production. Food Microbiol..

[5] Chand S.K., Nanda S., Mishra R., Joshi R.K. (2017). Multiple garlic (*Allium sativum* L.) microRNAs regulate the immunity against the basal rot fungus *Fusarium oxysporum* f. sp.. Cepae. Plant Sci..

[6] Pavlou G.C., Vakalounakis D.J., Ligoxigakis E.K. (2002). Control of root and stem rot of cucumber, caused by Fusarium oxysporum f. sp. radicis-cucumerinum, by grafting onto resistant rootstocks. Plant Dis..

[7] Li Z., Wang T., He C., Cheng K., Zeng R., Song Y. (2020). Control of Panama disease of banana by intercropping with Chinese chive (Allium tuberosum Rottler): Cultivar differences. BMC Plant Biol..

[8] Zhang H., Mallik A., Zeng R.S. (2013). Control of Panama disease of banana by rotating and intercropping with Chinese chive (*Allium tuberosum* Rottler): Role of plant volatiles. J. Chem. Ecol..

[9] Xu N., Wei M., Wang C., Shi W., Tian F.M., Wang X. (2013). Composition of Welsh onion (*Allium fistulosum* L.) root exudates and their allelopathy on cucumber sprouts and *Fusarium oxysporum* f. sp. cucumerinum. Allelop. J..

[10] Zuo G.W., Li C.Y., Li B., Wei Y.R., Hu C.H., Yang Q.S., Yang J., Sheng O., Kuang R.B., Deng G.M. (2015). The toxic mechanism and bioactive components of Chinese leek root exudates acting against Fusarium oxysporum f. sp. cubense, tropical race 4. Eur. J. Plant Pathol..

[11] Mylona K., Garcia-Cela E., Sulyok M., Medina A., Magan N. (2019). Influence of Two Garlic-Derived Compounds, Propyl Propane Thiosulfonate (PTS) and Propyl Propane Thiosulfinate (PTSO), on Growth and Mycotoxin Production by Fusarium Species In Vitro and in Stored Cereals. Toxins.

[12] Abdelrahman M., El-Sayed M., Sato S., Hirakawa H., Ito S.I., Tanaka K., Mine Y., Sugiyama N., Suzuki Y., Yamauchi N. (2017). RNA-sequencing-based transcriptome and biochemical analyses of steroidal saponin pathway in a complete set of Allium fistulosum-A. cepa monosomic addition lines. PLoS ONE.

[13] Nishioka T., Marian M., Kobayashi I., Kobayashi Y., Yamamoto K., Tamaki H., Suga H., Shimizu M. (2019). Microbial basis of Fusarium wilt suppression by Allium cultivation. Sci. Rep..

[14] Chand S.K., Nanda S., Joshi R.K. (2016). Regulation of miR394 in Response to Fusarium oxysporum f. sp. cepae (FOC) Infection in Garlic (*Allium sativum* L.). Front. Plant Sci..

[15] Zhang X., Wang H., Zhu W., Li W., Wang F. (2020). Transcriptome Analysis Reveals the Effects of Chinese Chive (*Allium tuberosum* R.) Extract on *Fusarium oxysporum* f. sp. radicis-lycopersici Spore Germination. Cur. Microbiol..

[16] Gow N.A.R., Latge J.P., Munro C.A. (2017). The Fungal Cell Wall: Structure, Biosynthesis, and Function. Microbiol. Spect..

[17] Arakane Y., Taira T., Ohnuma T., Fukamizo T. (2012). Chitin-related enzymes in agro-biosciences. Curr. Drug Targets.

[18] Desaki Y., Miyata K., Suzuki M., Shibuya N., Kaku H. (2018). Plant immunity and symbiosis signaling mediated by LysM receptors. Inn. Immun..

[19] Volk H., Marton K., Flajšman M., Radišek S., Tian H., Hein I., Podlipnik Č., Thomma B.P.H.J., Košmelj K., Javornik B. (2019). Chitin-binding protein of Verticillium nonalfalfae disguises fungus from plant chitinases and suppresses chitin-triggered host immunity. Mol. Plant Microb. Interact..

[20] Van Loon L.C., Rep M., Pieterse C.M.J. (2006). Significance of inducible defense-related proteins in infected plants. Ann. Rev. Phytopathol..

[21] Ali S., Ganai B.A., Kamili A.N., Bhat A.A., Mir Z.A., Bhat J.A., Tyagi A., Islam S.T., Mushtaq M., Yadav P. (2018). Pathogenesis-related proteins and peptides as promising tools for engineering plants with multiple stress tolerance. Microbiol. Res..

[22] Udaya Prakash N.A., Jayanthi M., Sabarinathan R., Kangueane P., Mathew L., Sekar K. (2010). Evolution, homology conservation, and identification of unique sequence signatures in GH19 family chitinases. J. Mol. Evol..

[23] Neuhaus J.M., Fritig B., Linthorst H.J.M., Meins F., Mikkelsen J.D., Ryals J. (1996). A revised nomenclature for chitinase genes. Plant Mol. Biol. Rep..

[24] Takenaka Y., Nakano S., Tamoi M., Sakuda S., Fukamizo T. (2009). Chitinase gene expression in response to environmental stresses in Arabidopsis thaliana: Chitinase inhibitor allosamidin enhances stress tolerance. Biosci. Biotechnol. Biochem..

[25] Yang H., Zhang T., Masuda T., Lv C., Sun L., Qu G., Zhao G. (2011). Chitinase III in pomegranate seeds (*Punica granatum* Linn.): A high-capacity calcium-binding protein in amyloplasts. Plant J..

[26] Ohnuma T., Numata T., Osawa T., Mizuhara M., Vårum K.M., Fukamizo T. (2011). Crystal structure and mode of action of a class V chitinase from Nicotiana tabacum. Plant Mol. Biol..

[27] Ohnuma T., Numata T., Osawa T., Mizuhara M., Lampela O., Juffer A.H., Skriver K., Fukamizo T. (2011). A class V chitinase from Arabidopsis thaliana: Gene responses, enzymatic properties, and crystallographic analysis. Planta.

[28] Ohnuma T., Umemoto N., Kondo K., Numata T., Fukamizo T. (2013). Complete subsite mapping of a “loopful” GH19 chitinase from rye seeds based on its crystal structure. FEBS Lett..

[29] Oliveira S.T., Azevedo M.I.G., Cunha R.M.S., Silva C.F.B., Muniz C.R., Monteiro-Júnior J.E., Carneiro R.F., Nagano C.S., Girão M.S., Freitas C.D.T. (2020). Structural and functional features of a class VI chitinase from cashew (*Anacardium occidentale* L.) with antifungal properties. Phytochemistry.

[30] Martínez-Caballero S., Cano-Sánchez P., Mares-Mejía I., Díaz-Sánchez A.G., Macías-Rubalcava M.L., Hermoso J.A., Rodríguez-Romero A. (2014). Comparative study of two GH19 chitinase-like proteins from Hevea brasiliensis, one exhibiting a novel carbohydrate-binding domain. FEBS J..

[31] Balu K.E., Ramya K.S., Radha A., Krishnasamy G. (2020). Structure of intact chitinase with hevein domain from the plant Simarouba glauca, known for its traditional anti-inflammatory efficacy. Int. J. Biol. Macromol..

[32] Broekaert W.F., Mariën W., Terras F.R., De Bolle M.F., Proost P., Van Damme J., Dillen L., Claeys M., Rees S.B., Vanderleyden J. (1992). Antimicrobial peptides from Amaranthus caudatus seeds with sequence homology to the cysteine/glycine-rich domain of chitin-binding proteins. Biochemistry.

[33] Taira T., Toma N., Ishihara M. (2005). Purification, characterization, and antifungal activity of chitinases from pineapple (*Ananas comosus*) leaf. Biosci. Biotechnol. Biochem..

[34] Cletus J., Balasubramanian V., Vashisht D., Sakthivel N. (2013). Transgenic expression of plant chitinases to enhance disease resistance. Biotechnol. Lett..

[35] Tobias P.A., Christie N., Naidoo S., Guest D.I., Külheim C. (2017). Identification of the Eucalyptus grandis chitinase gene family and expression characterization under different biotic stress challenges. Tree Physiol..

[36] Durechova D., Jopcik M., Rajninec M., Moravcikova J., Libantova J. (2019). Expression of Drosera rotundifolia Chitinase in Transgenic Tobacco Plants Enhanced Their Antifungal Potential. Mol. Biotechnol..

[37] Mohapatra R.K., Nanda S. (2018). In silico analysis of onion chitinases using transcriptome data. Bioinformation.

[38] Sun X., Zhu S., Li N., Cheng Y., Zhao J., Qiao X., Lu L., Liu S., Wang Y., Liu C. (2020). A Chromosome-Level Genome Assembly of Garlic (Allium sativum) Provides Insights into Genome Evolution and Allicin Biosynthesis. Mol. Plant.

[39] Huet J., Azarkan M., Looze Y., Villeret V., Wintjens R. (2008). Crystallization and preliminary X-ray analysis of a family 19 glycosyl hydrolase from Carica papaya latex. Acta Crystallogr. Sect. F Struct. Biol. Crystal. Commun..

[40] Misra B. (2015). Molecular Evolution and Functional Divergence of Chitinase Gene Family in Hevea brasiliensis Genome. Winnower.

[41] Hauenschild F., Favre A., Schnitzler J., Michalak I., Freiberg M., Muellner-Riehl A.N. (2017). Spatio-temporal evolution of Allium L. in the Qinghai-Tibet-Plateau region: Immigration and in situ radiation. Plant Diver..

[42] Khandagale K., Krishna R., Roylawar P., Ade A.B., Benke A., Shinde B., Singh M., Gawande S.J., Rai A. (2020). Omics approaches in Allium research: Progress and way ahead. PeerJ.

[43] Taylor A., Vagany V., Barbara D.J., Thomas B., Pink D.A.C., Jones J.E., Clarkson J.P. (2013). Identification of differential resistance to six *Fusarium oxysporum* f. sp. cepae isolates in commercial onion cultivars through the development of a rapid seedling assay. Plant Pathol..

[44] Chen J., Piao Y., Liu Y., Li X., Piao Z. (2018). Genome-wide identification and expression analysis of chitinase gene family in Brassica rapa reveals its role in clubroot resistance. Plant Sci..

[45] Jeffares D.C., Penkett C.J., Bähler J. (2008). Rapidly regulated genes are intron poor. Trends Genet..

[46] Zhou F., Guo Y., Qiu L.J. (2016). Genome-wide identification and evolutionary analysis of leucine-rich repeat receptor-like protein kinase genes in soybean. BMC Plant Biol..

[47] Passarinho P.A., de Vries S.C. (2002). Arabidopsis chitinases: A genomic survey. Arabidopsis Book.

[48] de Gerhardt L.B.A., Sachetto-Martins G., Contarini M.G., Sandroni M., de Ferreira R.P., de Lima V.M., Cordeiro M.C., de Oliveira D.E., Margis-Pinheiro M. (1997). Arabidopsis thaliana class IV chitinase is early induced during the interaction with Xanthomonas campestris. FEBS Lett..

[49] Rahman T.A., Oirdi M.E., Gonzalez-Lamothe R., Bouarab K. (2012). Necrotrophic pathogens use the salicylic acid signaling pathway to promote disease development in tomato. Mol. Plant-Microbe Interact..

[50] Veluthakkal R., Dasgupta M.G. (2012). Isolation and characterization of pathogen defence-related class I chitinase from the actinorhizal tree Casuarina equisetifolia. For. Pathol..

[51] Grover A. (2012). Plant Chitinases: Genetic Diversity and Physiological Roles. Crit. Rev. Plant Sci..

[52] Bartholomew E.S., Black K., Feng Z., Liu W., Shan N., Zhang X., Wu L., Bailey L., Zhu N., Qi C. (2019). Comprehensive Analysis of the Chitinase Gene Family in Cucumber (*Cucumis sativus* L.): From Gene Identification and Evolution to Expression in Response to Fusarium oxysporum. Int. J. Mol. Sci..

[53] Gao Y., Jia S., Wang C., Wang F., Wang F., Zhao K. (2016). BjMYB1, a transcription factor implicated in plant defence through activating BjCHI1 chitinase expression by binding to a W-box-like element. J. Exp. Bot..

[54] Gao Y., Zan X., Wu X., Yao L., Chen Y., Jia S., Zhao K. (2014). Identification of Fungus-Responsive Cis–Acting Element in the Promoter of Brassica Juncea Chitinase Gene, BjCHI1. Plant Sci..

[55] Verma V., Ravindran P., Kumar P.P. (2016). Plant hormone-mediated regulation of stress responses. BMC Plant Biol..

[56] Liu S., Kracher B., Ziegler J., Birkenbihl R.P., Somssich I.E. (2015). Negative regulation of ABA signaling by WRKY33 is critical for Arabidopsis immunity towards Botrytis cinerea 2100. Elife.

[57] Du M., Zhai Q., Deng L., Li S., Li H., Yan L., Huang Z., Wang B., Jiang H., Huang T. (2014). Closely related NAC transcription factors of tomato differentially regulate stomatal closure and reopening during pathogen attack. Plant Cell.

[58] Yang J., Duan G., Li C., Liu L., Han G., Zhang Y., Wang C. (2019). The Crosstalks Between Jasmonic Acid and Other Plant Hormone Signaling Highlight the Involvement of Jasmonic Acid as a Core Component in Plant Response to Biotic and Abiotic Stresses. Front. Plant Sci..

[59] Zhang C., Huang M., Sang X., Li P., Ling Y., Zhao F., Du D., Li Y., Yang Z., He G. (2019). Association between sheath blight resistance and chitinase activity in transgenic rice plants expressing McCHIT1 from bitter melon. Transgenic Res..

[60] Yang X., Yang J., Li H., Niu L., Xing G., Zhang Y., Xu W., Zhao Q., Li Q., Dong Y. (2020). Overexpression of the chitinase gene CmCH1 from Coniothyrium minitans renders enhanced resistance to Sclerotinia sclerotiorum in soybean. Transgenic Res..

[61] Kumar S., Stecher G., Tamura K. (2016). MEGA7: Molecular evolutionary genetics analysis version 7.0. molecular biology and evolution. Mol. Biol. Evol..

[62] Hu B., Jin J., Guo A.Y., Zhang H., Luo J., Gao G. (2015). GSDS 2.0: An Upgraded Gene Feature Visualization Server. Bioinformatics.

[63] Bailey T.L., Elkan C. (1994). Fitting a mixture model by expectation maximization to discover motifs in biopolymers. Proc. Int. Conf. Intell. Syst. Mol. Biol..

[64] Choi Y., Sims G.E., Murphy S., Miller J.R., Chan A.P. (2012). Predicting the functional eect of amino acid substitutions and indels. PLoS ONE.

[65] Babicki S., Arndt D., Marcu A., Liang Y., Grant J.R., Maciejewski A., Wishart D.S. (2016). Heatmapper: Web-enabled heat mapping for all. Nucl. Acids Res..

[66] Puchooa D. (2004). A simple, rapid and efficient method for the extraction of genomic DNA from lychee (*Litchi chinensis* Sonn.). Afr. J. Biotechnol..

[67] Leyronas C., Chrétien P.L., Troulet C., Duffaud M., Villeneuve F., Morris C.E., Hunyadi H. (2018). First report of Fusarium proliferatum causing garlic clove rot in France. Plant Dis..

[68] Sugui J.A., Deising H.B. (2002). Isolation of infection-specific sequence tags expressed during early stages of maize anthracnose disease development. Mol. Plant Pathol..

[69] Liu. M., Wu Z., Jiang F. (2015). Selection and validation of garlic reference genes for quantitative real-time PCR normalization. Plant Cell Tissue Organ Cul..

[70] Schwinn K.E., Ngo H., Kenel F., Brummell D.A., Albert N.W., McCallum J.A., Pither-Joyce M., Crowhurst R.N., Eady C., Davies K.M. (2016). The onion (*Allium cepa* L.) R2R3-MYB gene MYB1 regulates anthocyanin biosynthesis. Front. Plant Sci..

[71] Lescot M. (2002). PlantCARE, a Database of Plant Cis-Acting Regulatory Elements and a Portal to Tools for in Silico Analysis of Promoter Sequences. Nucleic Acids Res..

